# Supplementary material for: Dy4, Dy5, and Ho2 Complexes of an N3O2 Aminophenol Donor: A Dy5-µ3-Peroxide Single Molecule Magnet
Source: Int J Mol Sci. 2023 May 21;24(10):9061. doi: 10.3390/ijms24109061 (PMC10219328; doi:10.3390/ijms24109061)
Supplement: Supplementary file 1 [file ijms-24-09061-s001.zip › ijms-2348788-supplementary.pdf]

Supplementary Material

Dy_4, Dy_5 and Ho_2 Complexes of an N_3O_2 Aminophenol Donor: A Dy_5-μ_3-Peroxide Single Molecule Magnet

Julio Corredoira-Vázquez,[a,b] Paula Oreiro-Martínez,[a] Daniel Nieto-Pastoriza,[a] Ana M. García-Deibe,[a] Jesús Sanmartín-Matalobos,[a,c] Matilde Fondo,[a,*]

[a] Departamento de Química Inorgánica, Facultade de Química, Universidade de Santiago de Compostela, Campus Vida, 15782 Santiago de Compostela, Spain. E-mail: matilde.fondo@usc.es.
[b] Phantom-g, CICECO – Aveiro Institute of Materials, Department of Physics, University of Aveiro, 3810-193 – Aveiro, Portugal.
[c] Institute of Materials (iMATUS), Universidade de Santiago de Compostela, 15782 Santiago de Compostela, Spain

Table S1. Crystal data and structure refinement for **1**·2EtOH·H_2O-**3**·2.5H_2O S2

Table S2. Main bond distances (Å) and angles (º) for **1**·2EtOH·H_2O S3

Table S3. SHAPE v2.1. Continuous Shape Measures Calculation (c) 2013 Electronic Structure Group, Universitat de Barcelona S4

Table S4. Main bond distances (Å) and angles (º) for **2**·2H_2O S6

Table S5. Main bond distances (Å) and angles (º) for **3**·2.5H_2O S6

Figure S1. ^{1}H NMR spectrum for H_4L^r in DMSO-d_6 in the range 4.2-9.8 ppm S7

Figure S2. Comparative IR spectra for **2**·2H_2O and **4**·2EtOH·H_2O in the 1800-600 cm^{-1} region S7

Figure S3. Raman spectrum for **2**·2H_2O in the 600-1500 cm^{-1} region. S8

Figure S4. Comparative IR spectra for **2**·2H_2O and **5** in the 1800-600 cm^{-1} region S8

Figure S5. Comparison of X-powder diffractogram for the crystalline sample of **2**·2H_2O with the simulated one from single X-ray data S9

Figure S6. Comparison of X-powder diffractogram for the crystalline sample of **3**·2.5H_2O with the simulated one from single X-ray data S9

Figure S7. Cole-Cole plot for **2**·2H_2O in a zero dc field S10

Figure S8. Temperature dependence of χ''_M for **2**·2H_2O in a zero dc field at different frequencies S10

Figure S9. Left) frequency dependence of χ''_M for **1**·5H_2O at 3 K under different magnetic fields; right) dependence of the relaxation time with the field. S10

Figure S10. Temperature dependence of χ''_M in H_{dc} = 2000 Oe at two different frequencies for: left) **1**·5H_2O; right) **3**·2.5H_2O. S11

Figure S11. Left) frequency dependence of χ''_M for **2**·2H_2O at 6 K under different magnetic fields; right) dependence of the relaxation time with the field. S11

Table S1. Crystal data and structure refinement for **1**·2EtOH·H_2O-**3**·2.5H_2O

	**1**·2EtOH·H_2O	**2**·2H_2O	**3**·2.5H_2O
Empirical formula	$C_{65}H_{81}Cl_4Dy_4N_9O_{14}$	$C_{76}H_{70}Dy_5N_{16}O_{26}$	$C_{38}H_{39}Ho_2N_9O_{17.5}$
Molecular weight	2004.18	2435.98	1231.64
Crystal system	Monoclinic	Monoclinic	Monoclinic
Space group	*I*2/*a*	*C*2/*c*	*C*2/*c*
Wavelength (Å)	0.71073	0.71073	0.71073
Crystal size (mm^3)	0.14 x 0.13 x 0.13	0.08 x 0.05 x 0.03	0.450 x 0.189 x 0.032
Color, shape	Yellow, block	Orange, plate	Yellow, plate
T (K)	100	100	100
a (Å)	21.2306(16)	20.628(2)	31.657(2)
b (Å)	16.4877(10)	20.1771(17)	11.4734(6)
c (Å)	49.810(4)	23.198(2)	25.8050(16)
α (º)	90	90	90
β (º)	93.267(3)	99.533(4)	108.408(3)
γ (º)	90	90	90
Volume (Å^3)	17407(2)	9522.2(15)	8893.0(9)
Z	8	4	4
Absorpt. coef. (mm^{-1})	3.573	3.952	3.617
Reflections collected	402343	98996	195235
Independent reflections	26570 [R_{int} = 0.0711]	11335 [R_{int}= 0.0676]	13578 [R_{int}= 0.0647]
Data / restraints / param.	26570 / 0 / 928	11335 / 0 / 558	13578 / 2 / 625
Final R indices [I>2σ(I)]	R_1 = 0.0302<br>wR_2 = 0.0660	R_1 = 0.0301<br>wR_2 = 0.0624	R_1 = 0.0282<br>wR_2 = 0.0674
R indices (all data)	R_1 = 0.0480<br>wR_2 = 0.0731	R_1 = 0.0467<br>wR_2 = 0.0683	R_1 = 0.0402<br>wR_2 = 0.0732

Table S2. Main bond distances (Å) and angles (º) for **1**·2EtOH·H_2O

Dy1-O1	2.342(2)	Dy3-O1	2.437(2)
Dy1-O12	2.355(2)	Dy3-O32	2.462(2)
Dy1-O11	2.380(2)	Dy3-O11	2.327(2)
Dy1-O1W	2.386(3)	Dy3-O31	2.352(2)
Dy1-N11	2.546(3)	Dy3-O2W	2.386(3)
Dy1-N13	2.546(3)	Dy3-N32	2.563(3)
Dy1-N12	2.563(3)	Dy3-N33	2.612(3)
Dy1-Cl1	2.6960(8)	Dy3-N31	2.638(3)
Dy2-O21	2.318(2)	Dy3-Cl2	2.8532(8)
Dy2-O22	2.351(2)	Dy4-O32	2.286(2)
Dy2-O3S	2.438(2)	Dy4-O22	2.294(2)
Dy2-N21	2.499(3)	Dy4-O1	2.297(2)
Dy2-N22	2.499(3)	Dy4-O31	2.332(2)
Dy2-N23	2.544(3)	Dy4-O21	2.336(2)
Dy2-Cl32	2.6633(9)	Dy4-O12	2.349(2)
Dy2-Cl31	2.7546(9)	Dy4-O4S	2.369(2)
Dy1···Dy3	3.8693(4)	Dy2···Dy4	3.8259(3)
Dy1···Dy4	3.7890(3)	Dy3···Dy4	3.5410(3)
N11-Dy1-N12	63.199	O32-Dy3-N33	63.529
O12-Dy1-N11	164.668	O1-Dy3-N32	152.228
N22-Dy2-N23	65.759	O22-Dy4-O21	67.218
N23-Dy2-Cl21	149.467	O22-Dy4-O1	162.108

Table S3. SHAPE v2.1. Continuous Shape Measures Calculation (c) 2013 Electronic Structure Group, Universitat de Barcelona

Geometries Coordination number 7

JETPY-7	7 C3v	Johnson elongated triangular pyramid J7
JPBPY-7	6 D5h	Johnson pentagonal bipyramid J13
CTPR-7	5 C2v	Capped trigonal prism
COC-7	4 C3v	Capped octahedron
PBPY-7	3 D5h	Pentagonal bipyramid
HPY-7	2 C6v	Hexagonal pyramid
HP-7	1 D7h	Heptagon

Geometries Coordination number 8

ETBPY-8	13 D3h	Elongated trigonal bipyramid
TT-8	12 Td	Triakis tetrahedron
JSD-8	11 D2d	Snub diphenoid J84
BTPR-8	10 C2v	Biaugmented trigonal prism
JBTPR-8	9 C2v	Biaugmented trigonal prism J50
JETBPY-8	8 D3h	Johnson elongated triangular bipyramid J14
JGBF-8	7 D2d	Johnson gyrobifastigium J26
TDD-8	6 D2d	Triangular dodecahedron
SAPR-8	5 D4d	Square antiprism
CU-8	4 Oh	Cube
HBPY-8	3 D6h	Hexagonal bipyramid
HPY-8	2 C7v	Heptagonal pyramid
OP-8	1 D8h	Octagon

Geometries Coordination number 9

MFF-9	13 Cs	Muffin
HH-9	12 C2v	Hula-hoop
JTDIC-9	11 C3v	Tridiminished icosahedron J63
TCTPR-9	10 D3h	Spherical tricapped trigonal prism
JTCTPR-9	9 D3h	Tricapped trigonal prism J51
CSAPR-9	8 C4v	Spherical capped square antiprism
JCSAPR-9	7 C4v	Capped square antiprism J10
CCU-9	6 C4v	Spherical-relaxed capped cube
JCCU-9	5 C4v	Capped cube J8
JTC-9	4 C3v	Johnson triangular cupola J3
HBPY-9	3 D7h	Heptagonal bipyramid
OPY-9	2 C8v	Octagonal pyramid
EP-9	1 D9h	Enneagon

1·2EtOH·H_2O

Dy1

Structure [ML8]	ETBPY-8	TT-8	JSD-8	**BTPR-8**	JBTPR-8	JETBPY-8
	22.544,	8.607,	6.418,	**3.797**,	4.603,	26.599,

JGBF-8	**TDD-8**	SAPR-8	CU-8	HBPY-8	HPY-8	OP-8
8.619,	**3.346**,	4.325,	7.849,	7.951,	22.154,	34.851

Dy2

Structure [ML8]	ETBPY-8	TT-8	JSD-8	**BTPR-8**	JBTPR-8	JETBPY-8
	22.047,	12.094,	5.465,	**3.212**,	4.183,	26.802,

JGBF-8	**TDD-8**	SAPR-8	CU-8	HBPY-8	HPY-8	OP-8
12.963,	**2.270**,	3.633,	11.584,	14.022,	22.159,	32.870

Dy3

Structure [ML9]	**MFF-9**	HH-9	JTDIC-9	TCTPR-9	JTCTPR-9	**CSAPR-9**
	**1.522**,	9.720,	12.180,	3.169,	4.414,	**2.544**,

JCSAPR-9	CCU-9	JCCU-9	JTC-9	HBPY-9	OPY-9	EP-9
3.110,	6.910,	7.860,	15.304,	15.770,	22.449,	35.291

Dy4

Structure [ML7]	JETPY-7	JPBPY-7	**CTPR-7**	**COC-7**	PBPY-7
	17.621,	10.669,	**1.916**,	**1.214**,	7.239,

HPY-7	HP-7
18.940,	33.080

2·2H_2O

Dy1

Structure [ML9]	**MFF-9**	HH-9	JTDIC-9	TCTPR-9	JTCTPR-9	**CSAPR-9**
	**2.703**,	8.516,	11.653,	3.801,	4.882,	**2.814**,

JCSAPR-9	CCU-9	JCCU-9	JTC-9	HBPY-9	OPY-9	EP-9
3.832,	8.572,	9.907,	14.411,	18.263,	21.994,	37.134

Dy2

Structure [ML9]	**MFF-9**	HH-9	JTDIC-9	TCTPR-9	JTCTPR-9	**CSAPR-9**
	**2.805**,	8.292,	11.714,	3.637,	5.043,	**2.788**,

JCSAPR-9	CCU-9	JCCU-9	JTC-9	HBPY-9	OPY-9	EP-9
3.788,	8.555,	9.950,	14.535,	18.071,	21.712,	37.261

Dy3

Structure [ML8]	ETBPY-8	TT-8	JSD-8	**BTPR-8**	JBTPR-8	JETBPY-8
	22.968,	13.254,	5.593,	**5.570**,	6.423,	26.800,

JGBF-8	**TDD-8**	SAPR-8	CU-8	HBPY-8	HPY-8	OP-8
12.202,	**5.460**,	7.197,	12.466,	12.034,	23.141,	27.530

3·2.5H_2O

Ho1

Structure [ML9]	MFF-9	HH-9	JTDIC-9	**TCTPR-9**	JTCTPR-9	**CSAPR-9**
	1.809,	11.445,	11.026,	**1.480**,	3.099,	**1.214**,

JCSAPR-9	CCU-9	JCCU-9	JTC-9	HBPY-9	OPY-9	EP-9
1.975,	9.909,	10.999,	13.680,	19.597,	19.366,	36.809

Ho2

Structure [ML9]	**MFF-9**	HH-9	JTDIC-9	TCTPR-9	JTCTPR-9	**CSAPR-9**
	**1.931**,	7.708,	12.027,	2.848,	3.205,	**2.069**,

JCSAPR-9	CCU-9	JCCU-9	JTC-9	HBPY-9	OPY-9	EP-9
2.977,	8.299,	9.165,	13.934,	16.651,	21.585,	33.208

Table S4. Main bond distances (Å) and angles (º) for **2**·2H_2O

Dy1-O1	2.438(3)	Dy2-O1	2.295(2)	Dy3-O1	2.364(2)
Dy1-O2	2.312(2)	Dy2-O2	2.402(3)	Dy3-O2	2.370(3)
Dy1-O11	2.336(3)	Dy2-O21	2.322(3)	Dy3-O11	2.301(2)
Dy1-O12	2.274(3)	Dy2-O22	2.305(3)	Dy3-O21	2.323(3)
Dy1-O13	2.464(3)	Dy2-O23	2.496(3)	Dy1···Dy3	3.6317(4)
Dy1-O14	2.469(3)	Dy2-O24	2.461(3)	Dy2···Dy3	3.5923(4)
Dy1-N11	2.512(3)	Dy2-N21	2.514(4)	Dy1···Dy2	4.2636(6)
Dy1-N12	2.515(3)	Dy2-N22	2.483(3)		
Dy1-N13	2.557(3)	Dy2-N23	2.541(3)		
Dy1-N10	2.890(3)	Dy2-N20	2.895(4)		
O12-Dy1-O13	151.95(9)	O2-Dy2-N22	152.80(10)	O1[#1]-Dy3-O2	157.97(9)
O13-Dy1-O14	52.17(9)	O24-Dy2-O23	51.70(10)	O1-Dy3-O2	37.56(8)

[#1] = -x+1,y,-z+1/2

Table S5. Main bond distances (Å) and angles (º) for **3**·2.5H_2O**.**

Ho1-O11	2.314(2)	Ho2-O11	2.346(2)
Ho1-O21	2.350(2)	Ho2-O21	2.306(2)
Ho1-O12	2.385(2)	Ho2-O22	2.355(2)
Ho1-O1W	2.401(2)	Ho2-O2W	2.390(2)
Ho1-O14	2.459(2)	Ho2-O23	2.436(2)
Ho1-O13	2.491(2)	Ho2-O24	2.504(2)
Ho1-N12	2.504(2)	Ho2-N22	2.528(3)
Ho1-N11	2.531(3)	Ho2-N23	2.529(3)
Ho1-N13	2.535(2)	Ho2-N21	2.529(3)
Ho1···Ho2	3.8091(2)		
O14-Ho1-O13	51.79(8)	O23-Ho2-O24	51.88(7)
O11-Ho1-N13	145.88(8)	O22-Ho2-O24	148.43(7)

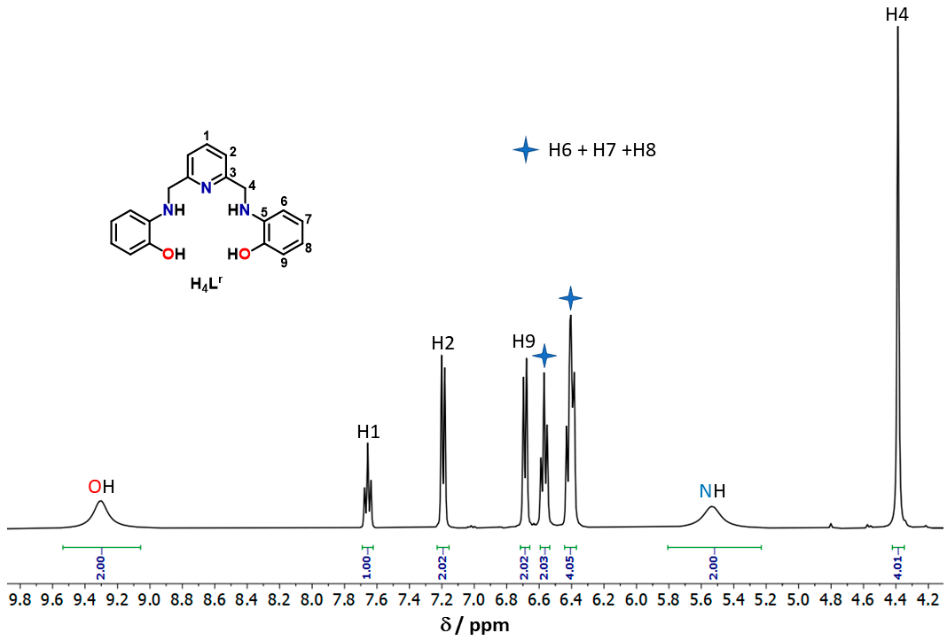

Figure S1. ^1H NMR spectrum for H_4L^r in DMSO-d_6 in the range 4.2-9.8 ppm.

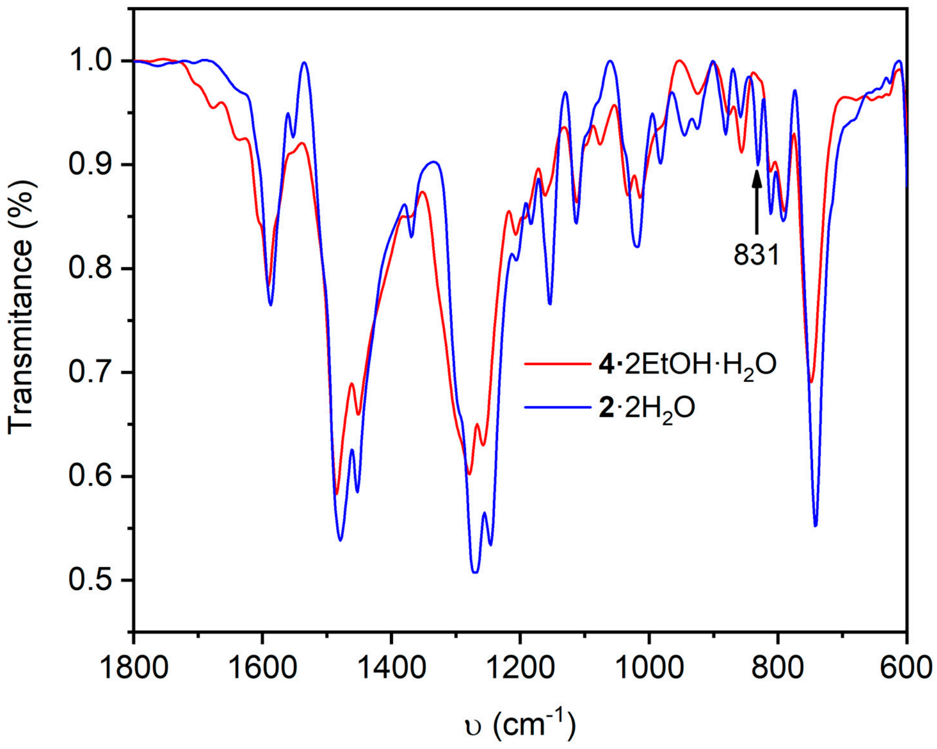

Figure S2. Comparative IR spectra for **2**·$2H_2O$ and **4**·2EtOH·H_2O in the 1800-600 cm^{-1} region.

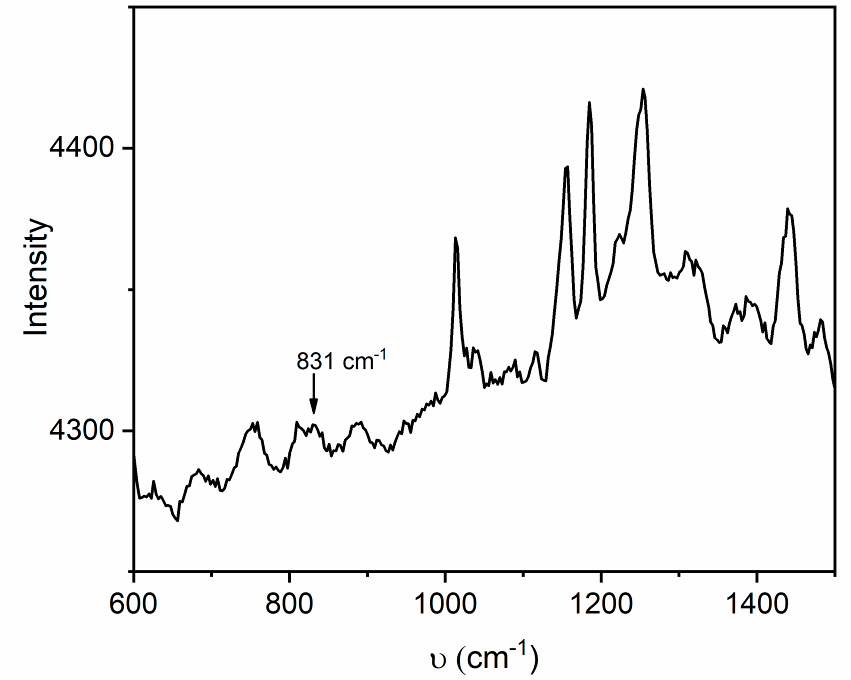

Figure S3. Raman spectrum for **2**·2H_2O in the 600-1500 cm^{-1} region.

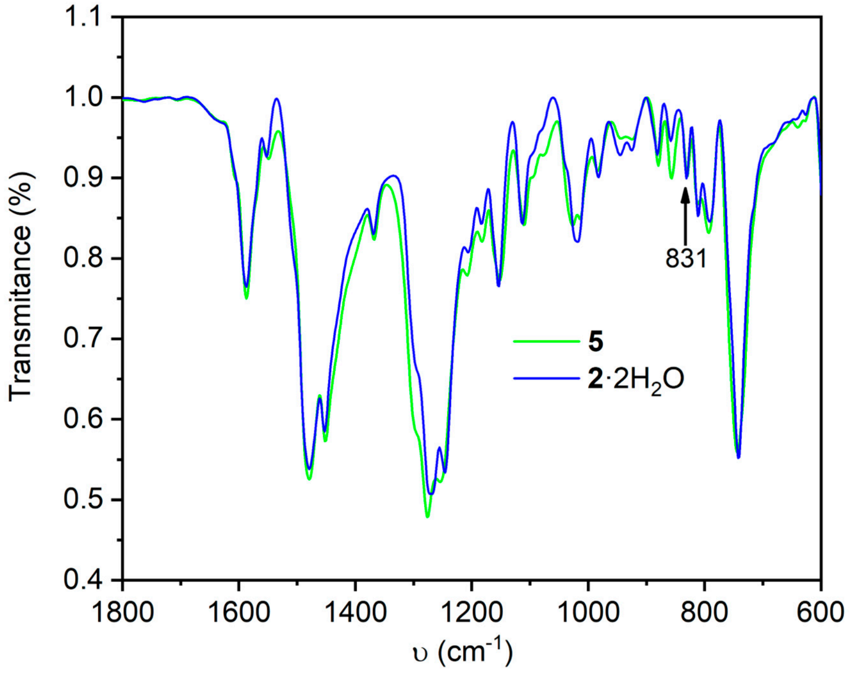

Figure S4. Comparative IR spectra for **2**·2H_2O and **5** in the 1800-600 cm^{-1} region.

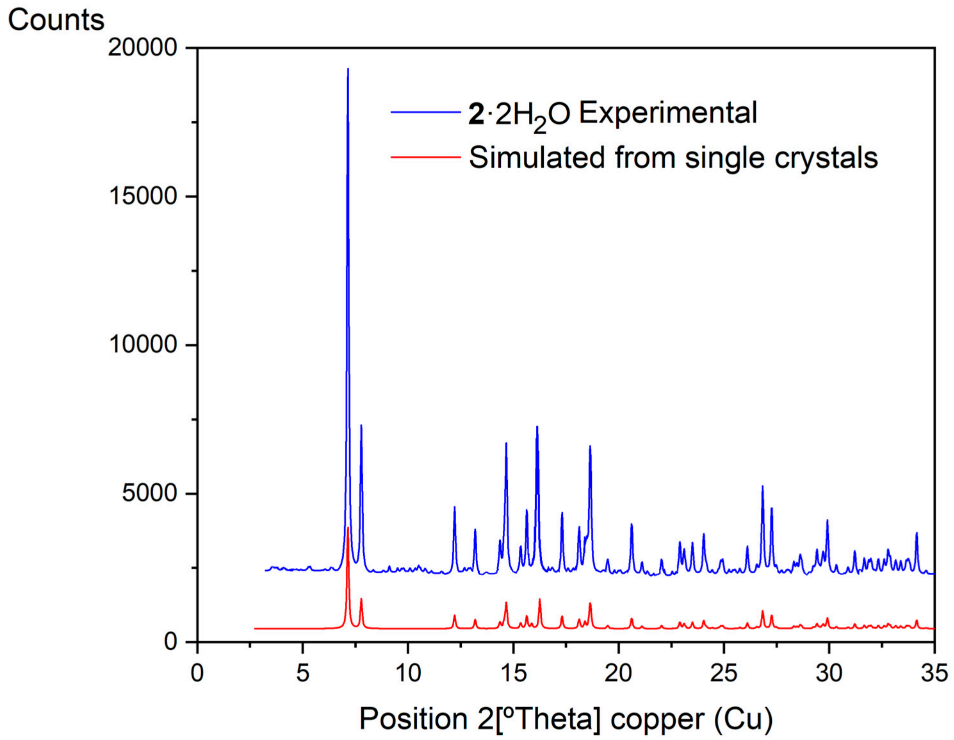

Figure S5. Comparison of X-powder diffractogram for the crystalline sample of **2**·2H_2O with the simulated one from single X-ray data.

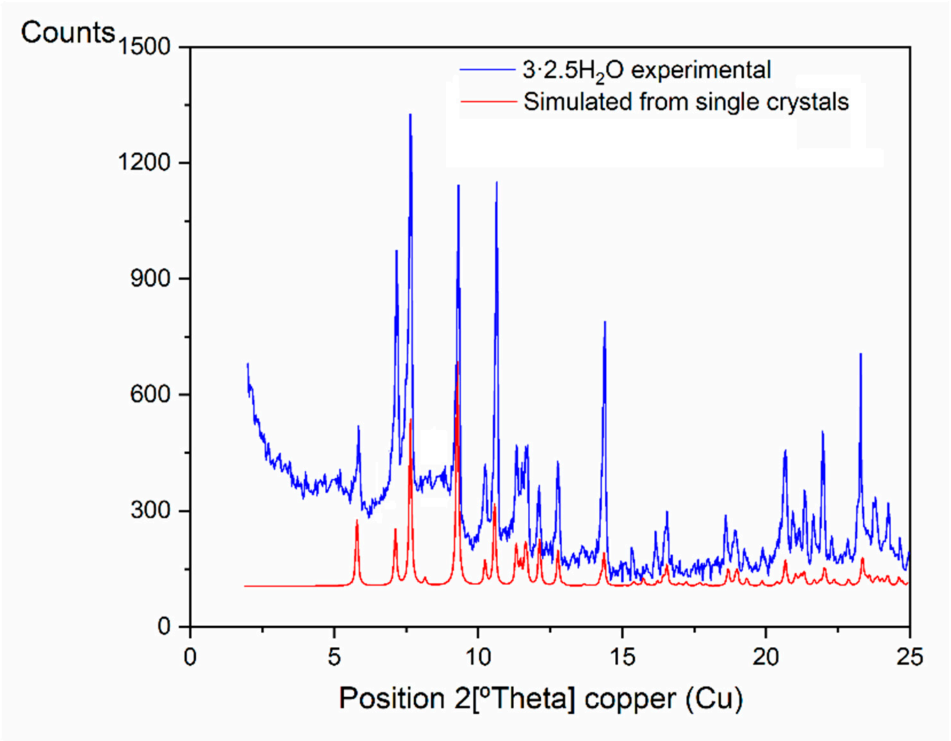

Figure S6. Comparison of X-powder diffractogram for the crystalline sample of **3**·2.5H_2O with the simulated one from single X-ray data.

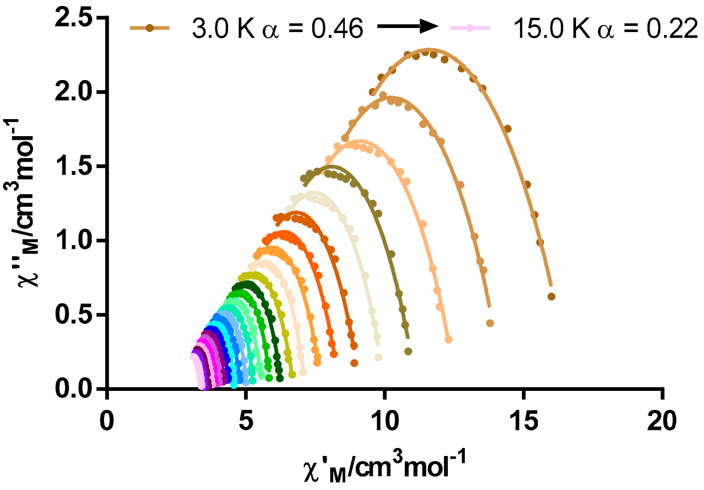

Figure S7. Cole-Cole plot for **2**·$2H_2O$ in a zero dc field

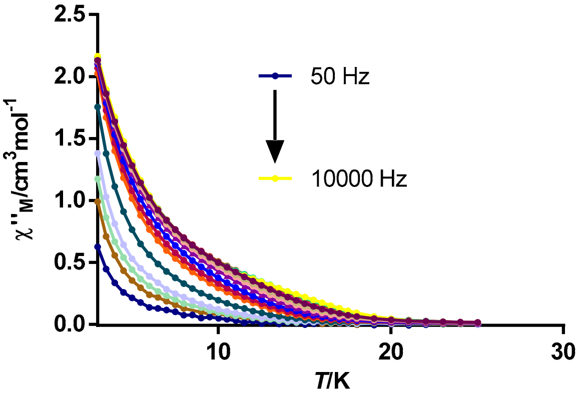

Figure S8. Temperature dependence of χ''_M for **2**·$2H_2O$ in a zero dc field at different frequencies.

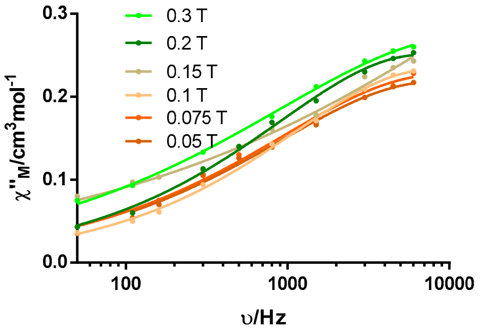

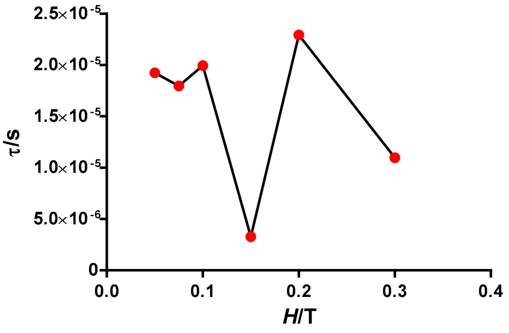

Figure S9. Left) frequency dependence of χ''_M for **1**·$5H_2O$ at 3 K under different magnetic fields; right) dependence of the relaxation time with the field.

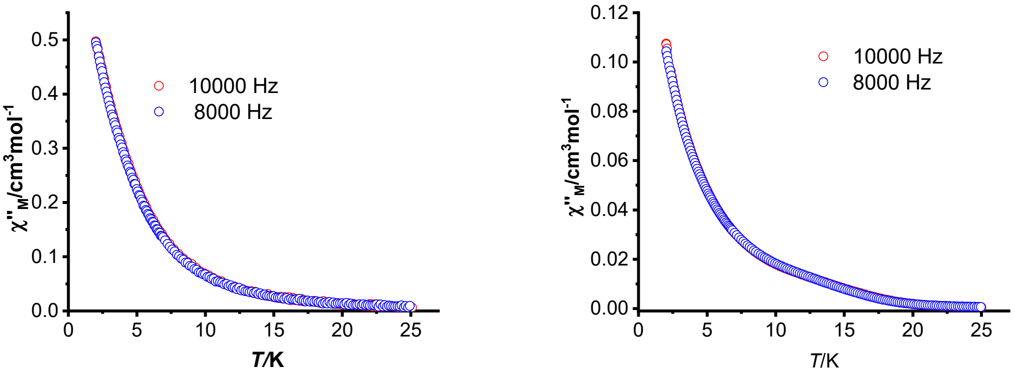

Figure S10. Temperature dependence of χ''_M in H_{dc} = 2000 Oe at two different frequencies for: left) **1**·5H_2O; right) **3**·2.5H_2O.

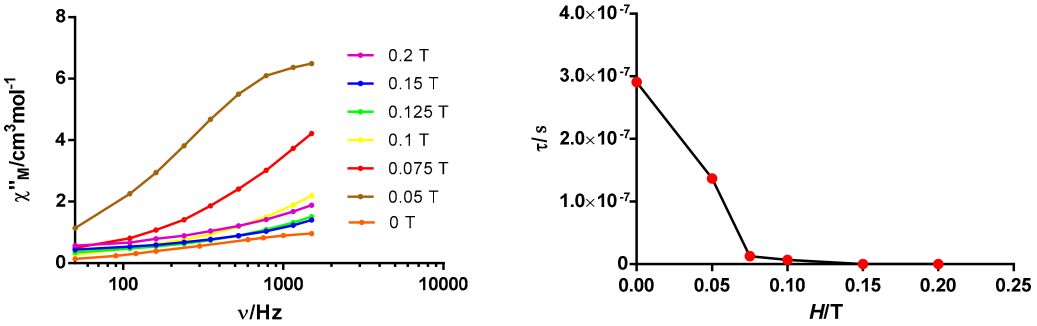

Figure S11. Left) frequency dependence of χ''_M for **2**·2H_2O at 6 K under different magnetic fields; right) dependence of the relaxation time with the field.
